# The Early Years of Parenthood and Home‐Start Home Visiting: An Interpretative Phenomenological Analysis

**DOI:** 10.1002/jcop.70048

**Published:** 2025-10-14

**Authors:** Martha Burlingham, Dr Katerina Kantartzis

**Affiliations:** ^1^ Department of Psychology University of Gloucestershire Cheltenham England

**Keywords:** child, community support, family, infants, parenting, parents, preschool, qualitative research

## Abstract

To explore the lived experience of the early years of parenthood and the effect of Home‐Start home visiting, in light of the impact from the polycrisis on parenthood and the critical early years. Four semi structured interviews were conducted with five parents (three mothers and one couple [mother and father]) who had accessed Home‐Start Gloucestershire home visiting. The interviews were analyzed using Interpretative Phenomenological Analysis. There were four Group Experiential Themes, each with two subthemes, showing a transformative journey. Parents navigated contrasting realities and multifaceted layers of parenthood. Accepting help led to meaningful connections with Home‐Start creating a butterfly effect for families. The early years of parenthood are complex and emotive. It is difficult yet important for parents to access support, helped by trusting relationships with early intervention services. This qualitative research informs early intervention services highlighting their criticalness on parent experience.

## 1 Introduction

### 1.1 The Early Years of Life

The early years of a child's life starting from conception is recognized as central for promoting healthy physical, emotional, social, and intellectual development (Shonkoff & Phillips 2000). During the first 1001 critical days from conception, the brain is growing and developing extremely quickly meaning it is more prone to outside influence than any other time in the lifecourse (Leach 2017; Leadsom et al. 2013). Optimal brain development during the early years gives a new child the best possible start in life that impacts on the rest of their life (Leach 2017; Sheridan and Nelson 2009).

### 1.2 The Complexities of the Transition to Parenthood

Parents and caregivers are the chief architects of early childhood development, shaping the experiences that build children's brains. Parenting involves the provision of nurturing care throughout childhood to help prepare children to live in society, form relationships, learn, work and thrive (UNICEF 2023). Providing this nurturing care takes time, resource and services, all of which can be in short supply for parents and caregivers around the world due to poverty, deprivation, conflict, other crises, lack of access to quality services, or stretched thinly due to poor mental health, stress and the struggles with work and family life balance (UNICEF 2023).

The transition to parenthood can be complex: it is life altering and highly rewarding yet highly challenging (Cowan and Cowan 2000; Feeney 2001). Although it can enhance parent's wellbeing, it can also be a detriment, with changes in life roles, fatigue, financial burden, work‐family conflict amongst others, all contributing to stress (Cowan and Cowan 2000; Kohn et al. 2012). Research has consistently demonstrated the stress of the transition to parenthood and its bringing of more profound changes than any other developmental stage of the family life cycle (Cowan and Cowan 1995; Priel and Besser 2002). Parent wellbeing can have significant implications for child wellbeing, their development outcomes and society (Mackler et al. 2015; Turney 2011). If parenting stress is high, it could lead to a more chaotic family environment which can impact children's wellbeing (Bagner et al. 2009). This could lead to lower self esteem and higher anxiety for children (Fiese and Winter 2010). A study conducted in the United States (US), found that children living in homes where caregivers reported higher levels of parenting stress were more likely to experience four or more Adverse Childhood Experiences (ACEs) by the age of 18. Therefore, lowering parenting stress through parenting interventions could decrease the level of childhood trauma experienced by a child or may lessen one type of stress in a home where many other stressors exist (Crouch et al. 2019).

### 1.3 The Polycrisis and Families

In 2022 the world experienced a series of major crises including a pandemic, a major war in Europe, energy crisis, rising inflation and food insecurity. These multiple simultaneous crises have been strongly interdependent; termed a polycrisis (UNICEF 2023). We continue to be impacted by the polycrisis and it continues to impact on children's lives (UNICEF 2023). Research has found that parental stress increased significantly due to the impact of the COVID‐19 pandemic. Parental wellbeing was an important target point for interventions addressing the consequences of the pandemic (Calvano et al. 2022). It is recognized that parenting is too big a task for parents and caregivers to do alone and that support is needed to help give children the best possible start in life (UNICEF 2023). Supporting families is, consequently, an investment in the future of children's health (Crouch et al. 2019).

### 1.4 The Importance of Early Intervention

The importance of helping families early is recognized in national safeguarding guidance in the United Kingdom (UK) as well as its importance during the early years of life (HM Government 2021). Identifying and addressing need early can increase protective factors and impact on family wellbeing (EIF 2020). Research has shown that a variety of early interventions in the first 5 years of life that attend to specific needs of children and families in a variety of circumstances have had positive results (Center on the Developing Child 2007). Understanding the impact of targeted early intervention policies on the life‐long development of children is an increasingly important focus of policymakers. One potential for focus is the welfare improvement for parents, benefitting their wellbeing directly and also indirectly improving child wellbeing and development (Doyle et al. 2017).

### 1.5 Home‐Start Home Visiting

Home visiting is one such early intervention used, where a volunteer or professional visits the family in their home to provide emotional and practical support with the aim of promoting the healthy development of children, enhance parenting skills, and improving family wellbeing (Finello et al. 2016). Home‐Start is a charity that provides home visiting. Operating globally in 22 countries, Home‐Start tailors its services to meet local needs while maintaining a central mission: to strengthen families by providing emotional and practical support. Home‐Start uses a community based model, with volunteers going into the home (Home‐Start Worldwide 2024).

The Home‐Start home visiting intervention in the UK provides individualized, volunteer‐based support to families with children under 5 years old, particularly those facing challenges such as poverty, isolation, mental health issues, and disabilities (Home‐Start 2025). Volunteers typically visit families in their homes for 2–3 h per week, with the frequency and duration of visits determined collaboratively between the family, volunteer, and Home‐Start coordinator based on each family's specific needs and circumstances. Rather than following a standardized approach, Home‐Start operates using a flexible, family‐led approach where volunteers provide practical and emotional support tailored to what each family identifies as their priorities. This may include helping with daily routines, providing a listening ear, accompanying families to appointments, or supporting parenting confidence. Volunteers are recruited from the local community and are often parents themselves who can relate to the challenges families face. All volunteers complete a comprehensive preparation course that covers child development, family dynamics, confidentiality, safeguarding, and communication skills, followed by ongoing supervision and support from trained Home‐Start coordinators. The length of support varies according to family needs, typically ranging from a few months to a year, with the goal of building family confidence and connections to enable sustainable positive change (Home‐Start 2025).

The importance of Home‐Start services has become increasingly apparent in the wake of the COVID‐19 pandemic, which exacerbated existing struggles and introduced new challenges for families, including rising rates of domestic abuse, mental illness, and child poverty (Home‐Start 2021).

### 1.6 Home‐Start Research

While Home‐Start evaluates its services and provides impact reports at local and national levels (Home‐Start 2014, 2017, 2019, 2021), these reports primarily focus on quantitative measures, such as pre‐ and post‐intervention questionnaire scores. Qualitative feedback and quotations from parents are included in these reports to reflect key experiences and information but no in depth qualitative analysis was conducted. There have been other quantitative studies exploring Home‐Start home visiting conducted in collaboration with universities and research centers, which have shown beneficial outcomes to parent and child wellbeing (Hermanns et al. 2013; Kenkre and Young 2013; Warner 2019).

Furthermore, there are two mixed‐methods evaluations of Home‐Start, which combined experimental designs with qualitative interviews with parents which showed that parents valued Home‐Start support (Barnes et al. 2006; McAuley et al. 2004). Parents expressed improvements to their mental health because of Home‐Start (McAuley et al. 2004). Qualitative studies (Oakley et al. 1998; Frost et al. 2000; McAuley et al. 2004; MacPherson et al. 2010) have also shown that a number of parents value Home‐Start's support. For example, mothers reported feeling less depressed, isolated, lonely and pressured with some expressing better relationships with their children and partners (Bagihole 1996). In MacPherson et al's (2010) study, 23 mothers all made at least one positive comment about Home‐Start support. However, some parents reported difficulties with support such as problems with the administration of the schemes and how the support was withdrawn, and mismatches between the families and the volunteers. These qualitative findings suggest that some parents may find the support more valuable than others yet with an overarching theme that the support from Home‐Start was positive.

Home‐Start Hertfordshire in the United Kingdom evaluated a newly developed service to support women at risk of perinatal anxiety and depression, visited in their homes by trained volunteers. Volunteer feedback and interviews with coordinators indicated that women found the service constant, reliable and an unconditional source of support, showing the value of the new service for the mothers (Burn and Almack 2018). Although these qualitative studies show the value of Home‐Start home visiting, there is a gap in the research in exploring in depth the complex and nuanced experiences of parents and what this could mean in informing Home‐Start home visiting, particularly given that previous research has found there is a variation in the value attributed to Home‐Start.

### 1.7 Wider Home Visiting Research

In the wider home visiting literature, home visiting programs have also predominantly relied on quantitative evaluations to assess the effectiveness of these interventions in enhancing family and child outcomes (Butler et al. 2020). While these evaluations are essential for demonstrating efficacy and justifying broader program implementation, they may not capture the full complexity of participants' experiences (Butler et al. 2020). Understanding early interventions on a personal level is critical for adapting services to meet the unique needs of diverse families (Furlong and McGilloway 2012; Holtrop et al. 2014). By exploring parents' perceptions and experiences through qualitative methods, researchers can illuminate the key aspects of change that make these interventions meaningful and impactful for families (Kane et al. 2007). Previous research supports the value of qualitative inquiry in measuring the impact of parent support programs like home visiting (Butcher and Gersch 2014), highlighting how qualitative methods can complement quantitative findings by offering a richer dimension of understanding about parents' experiences (Butcher and Gersch 2014; Levac et al. 2008).

### 1.8 The Current Study

In Gloucestershire, Home‐Start has expanded its reach by forming a consortium that offers various services, including home visits, antenatal and postnatal groups, perinatal mental health support, and a Dad Matters scheme. The aim of this qualitative study was to explore parents' experiences of the early years of parenting and their experiences of Home‐Start Gloucestershire's home visiting services. Specifically, three research questions were addressed:

− How do parents make sense of and navigate their experiences of the early years of parenthood?
− What were parents' experiences of Home‐Start Gloucestershire home visiting services?
− What were parents' experiences of navigating parenthood like before, during and after accessing Home‐Start Gloucestershire's home visiting services?

## 2 Methods

### 2.1 Design

Interpretative Phenomenological Analysis (IPA) aims to make sense of participants' life experiences through three approaches: phenomenology where lived experiences are explored, hermeneutics which interprets meaning and what experiences mean to individuals and idiographic where focus is on individual cases and each individual's unique perspective. IPA is often used for research on transition (Smith et al. 2022). It was deemed the most appropriate method for making sense of how parents make sense of the major transition of becoming and being a parent and accessing support. Parents' experiences are diverse and shaped by various factors, including socioeconomic background, cultural context, and personal circumstances, all of which can significantly influence their interactions with support services. IPA is particularly well‐suited for exploring these diverse experiences, as it emphasizes how individuals make sense of key life transitions, such as parenthood (Smith et al. 2022). By using IPA, researchers can uncover the emotional depth and personal meanings that parents attach to their experiences with Home‐Start, leading to richer insights that quantitative evaluations may overlook. This qualitative approach aligns with Home‐Start's overarching mission to provide empathetic, family‐centered support that acknowledges the complexities of parenthood.

### 2.2 Participants

The participants consisted of parents—mothers, fathers, or both—who had accessed Home‐Start Gloucestershire's home visiting service for any reason, with their most recent engagement occurring in 2022 or 2023. Purposive sampling was employed in this study, with Home‐Start Gloucestershire staff assisting in identifying and reaching out to families who might be interested in participating in the research. This approach was essential, given the specific focus on families who had used Home‐Start's services and the unique challenges they faced. The Home‐Start staff had established relationships with the families and were familiar with their circumstances, enabling them to identify families that met the criteria for participation.

The criteria for selection included families who had engaged with Home‐Start's home visiting service in the past year and demonstrated a willingness to share their experiences. Additionally, the staff considered the families' current emotional and psychological readiness, as participation in research could involve discussing potentially sensitive topics related to parenting and support services. Given the potentially vulnerable nature of these families, the Home‐Start staff were well‐positioned to determine which families were in a suitable place to engage meaningfully in the study. This eligibility criteria is narrow to have a homogenous sample, typical of IPA methodology to explore phenomena from participant's points of view and how they make sense of it (Alase 2017).

Initially, six families were approached based on these criteria, and ultimately, four of these families agreed to participate. Following guidance by Smith et al. (2022), this was still an appropriate number of participants for IPA. Their contact details were passed to the first author (MB) and a Participant Information Sheet detailing the study was provided. If parents were interested in taking part, a time and date were agreed with a choice of meeting in their own home, university campus or MS Teams. Ethics approval for this study was received.

Before starting the interview, participants were given an overview of the study (based on the Participant Information Sheet). Their rights to withdraw were provided and they were reassured that they could pause or stop the interview at any point. A consent form was provided to sign. After the interview, a debriefing sheet was provided. The interviews could raise emotive subjects and information on where to access further support was given in the debriefing sheet. The interviewer (MB) stayed mindful of any cues regarding the participant's emotional state throughout the interview in case a break or time was needed for the participants.

### 2.3 Data Collection

There were five participants with four semi‐structured interviews undertaken by MB (one‐to‐one interviews with three mothers and one interview with a couple (mother and father)). Two interviews were online via MS Teams and two interviews were in the parents' home. Interviews were recorded via MS Teams if online or by Dictaphone if face to face. The interview was up to 1 h in length. The questions focused on parent's lived experiences of becoming/being a parent during the early years before, during and after accessing Home‐Start Gloucestershire and their lived experiences of accessing Home‐Start Gloucestershire home visiting. A guideline of questions was used but the interviewer was also guided by the participant and asked questions based on their responses, following their individual journey. MB debriefed with KK and a reflective log was kept my MB after each interview documenting their own thoughts, feelings, and reflections.

### 2.4 Analysis

Interpretative Phenomenological Analysis (IPA) was used to analyze the interviews. Firstly, recorded interviews were transcribed in vertabim. This was done via human transcription without software to enable the researcher to fully immerse themselves in the participant's life world. This allowed a deep understanding of the interview and helped with later analysis of “knowing” the transcript (Halcomb and Davidson 2006).

After transcription, each interview transcript was analyzed separately following the IPA process. The transcript was read and reread to become familiar. Next, exploratory notes were made. These are initial thoughts and observations made by the researcher regarding the participant's experiences. Next, experiential statements were formed to summarize important sections of exploratory notes whilst still being grounded in the original text. This is the process of making sense of how the participant is making sense of their lived experiences. Experiential statements were clustered together to make personal experiential themes. This process was repeated for each participant. Cross‐case analysis was then conducted where the data was compared across the four cases. Similarities and differences were analyzed helping to further understand the individual participant and understand the group. The process of IPA was iterative.

Analysis was conducted by MB. Credibility checks were completed by the second author (KK). They reviewed the logged process conducted by MB. Transcripts were checked to ensure initial and later themes reflected the data. MB kept a reflective log throughout the process from the interviews to the analysis. MB and KK discussed their reflections and observations. MB's own circumstances were discussed and how this may interact with the analysis‐ particularly given that IPA is about how the researchers make sense of how the participants make sense of their experiences‐ bringing awareness surrounding bias and how the author was interpretating the data. MB is a mother with twins who were age four during the analysis process. Interpretive phenomenology shows that we as people are so enmeshed in our world that the researcher cannot and also should not hold back their prior understanding or engagement of the subject being studied. The depth of involvement of researchers means that it would confirm credibility (Reiners 2012). The researchers take this stance yet also remained aware of how their own experiences were interacting with the interpretation.

## 3 Results

Participant details are presented in Table 1. All participants have been pseudonymised with parents in the same family given pseudonyms starting with the same letter. On reflection, it would have been preferred to ask families to choose their pseudonyms, as researchers are mindful of the meaning of names to individuals. However, the researchers chose the pseudonyms from an assortment of names that have been popular in the last few decades

**Table 1:** Participant details.

Parent name	Number of children in the home at the time of home visiting	Length of time accessing home‐start home visiting	Interview method
Amanda (mother)	One daughter	9 months	In person in parent's home
Christina (mother)	Two daughters	Accessed home visiting three times over the course of approximately 3 years. Time one: 6 months Time two: 6 months Time three: 6 months	MS Teams
Sophie (mother) and Steven (father)	Three sons (older sibling and twins)	10 months	In person in parents' home
Jennifer (mother)	One son	3 months	MS Teams

**Figure 1:**
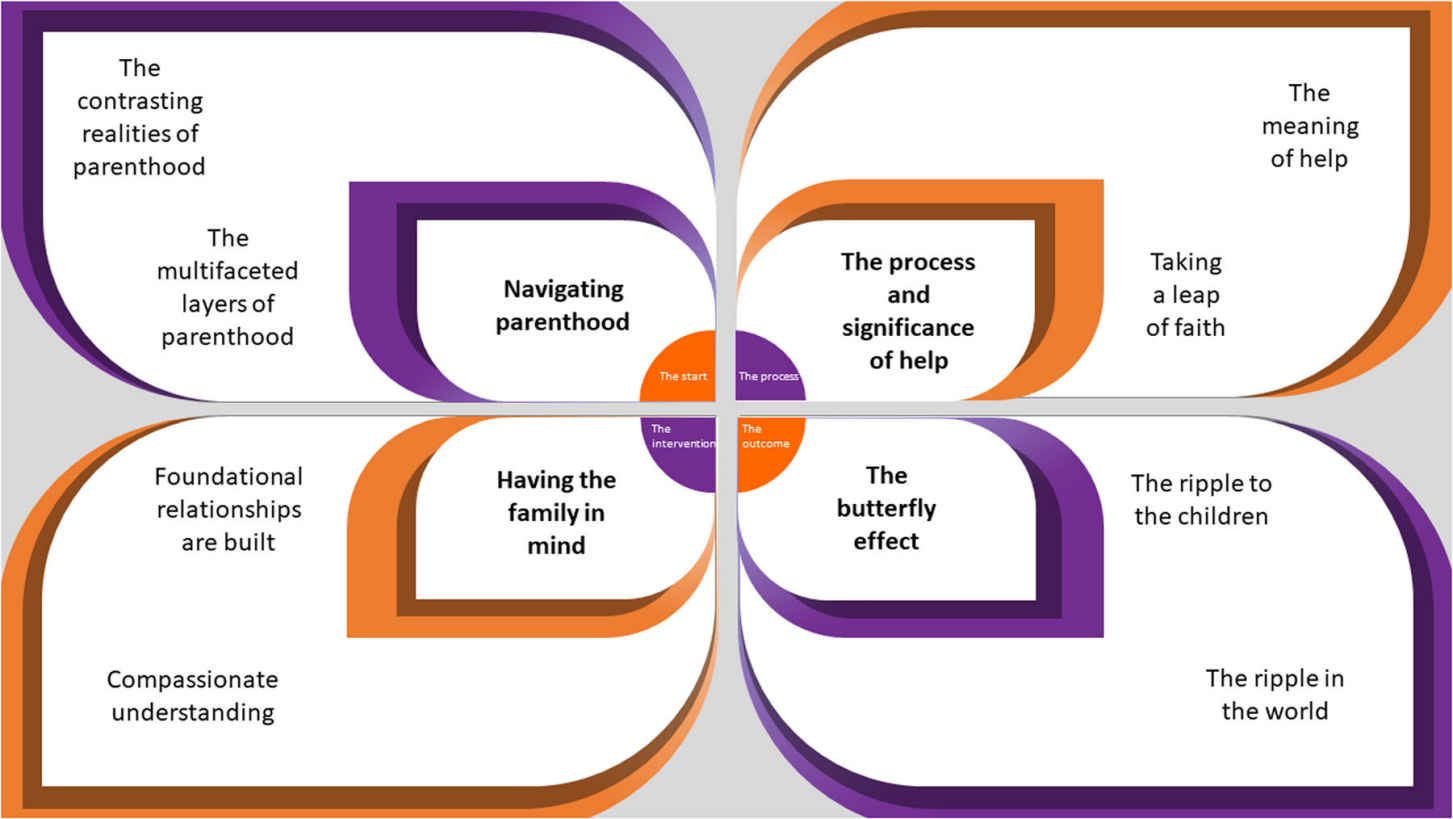
IPA Group Experiential Themes and subthemes. A figure showcasing the Group Experiential Themes in the four inner wings and the related subthemes in the outer wings. The circle in the middle demonstrates the journey for the parents through the themes from the start of the early years of parenthood navigating its realities, the process of accepting help, accessing home visiting (the intervention) and the outcome and impact of accessing home visiting.

Four Group Experiential Themes were identified, each with two subthemes, showing the parents' transformational journey before, during and after Home‐Start home visiting (see Figure 1). The Group Experiential Themes, subthemes and indicative quotes are presented in Table 2.

**Table 2:** Group experiential themes, subthemes, and quotes.

Group experiential themes	Subthemes	Quotes
Navigating parenthood	The contrasting realities of parenthood	**Jennifer** *: “Um it was yeah it was challenging but very but very rewarding at the same time so”* **Steven** *: “…we faced the fact of life possibly without children and um we weren't really okay with that, you particularly had always longed to be a mother”* **Sophie** *: “Mm”* **Steven** *: “I was like oh it will be fine. But then you taste it you enjoy it and you're like this is incredible such a privilege and joy er to not have it would it's very sad… Yeah, it's the greatest privilege of our life isn't it.”* **Sophie** *: “Yeah it is amazing and I think even the hard times it just makes up for it the fact that we're getting to do this we're getting to be a family and we're getting to experience it and just yeah the joys and the hard times.”* **Sophie** *: “it was hard going you were shocked weren't you I don't think you were quite“* **Steven** *: “Oh my goodness yeah I was not prepared I'm an optimist and I thought I would take it in my stride. I thought he would fit into life which really didn't happen at all”* **Amanda** *: “And then when I got pregnant. The first thing I thought was I'm gonna die. I can't give birth…Like but she's my absolute world. I don't know. I can't remember life before having her.”* **Christina** *: “there's a village that didn't really have people that had newborn babies so umm yeah, especially being pregnant on my own didn't really know anyone couldn't drive, couldn't go anywhere. Um so yeah, it was pretty, pretty difficult start really”* **Jennifer** *: “Yes. So I I experienced postnatal after having my first um because being a new mum and everything, it was very daunting and I didn't really have support from um family or anything um”*
	The multifaceted layers of parenthood	**Christina** *: “…I just think all this is sort of normal being a mum feeling like this. I've always suffered with anxiety anyway and um a bit of depression, umm but just another um another layer I suppose.”* **Amanda** *: “It's been hard cuz during COVID like most of our life, like, I have to be the one person that had to have a baby during that…And my first child. And no one got to hold her. I was hoping that my birth would be give birth a couple of days, go to my mum…And it weren't like that. It was four months later, go to my mum. Yeah. So you didn't like it felt a bit robbed”* **Christina** *: “unfortunately I had spent endured six months of hell living with him um because I wasn't able to leave the house umm and so that's when it got a lot lot worse umm and that's where the majority of my PTSD comes from really is when we were living in the same household.”* **Sophie** *: “. . despite all our wanting children I was like I do not want two at the same time so it took me a long time to come round to it”* **Steven** *: “See I didn't have that perspective because I was just like well we've done it for one, just do the same thing again but for two…She was like you have no idea, it doesn't work like that”* **Sophie** *: “(laughs as Steven speaks)”* **Steven** *: “But she was right it didn't work like that”* **Steven** *: “When the NICU stay as well which we came to it with twins was challenging, trying to care for [oldest child] here, feeling guilty for not being in NICU with the twins and all that kind of stuff”* **Steven** *: “We we were I mean we were surviving and doing well in terms of the kids were happy you know [oldest child] wouldn't have said I was neglected around that time or anything, there was fun in the house and we did all we could, but we were privately our heads were just above water so we didn't ever do anything around the house did we we had no capacity for that, you had no break whatsoever, there was no free time”* **Jennifer** *: “Yeah. When he wants something but you don't know what he wants because he can't communicate so, yeah.”* **Interviewer** *: “Yeah. How does that make you feel when he has a meltdown?”* **Jennifer** *: “Um really anxious and just really like you know I do I do feel a bit not helpless but just it's really difficult because like I don't know what you want (laughs)”* **Christina** *: “I found it hard work because I had a child who was just over two who was very clingy and needy and obviously her world was knocked upside down um and she hated her sister for about six months”*
The process and significance of help	The meaning of help	**Christina** *: “it's quite difficult when you, you know, you're to ask for help anyway generally, being a new mum, old mum, whatever um I think it's quite difficult to to to take that help as well. Um so I think maybe for (breathes out) new mums I think it's even worse if you don't have anyone around you”* **Christina** *: “and there is that sort of not stigma. I wouldn't call it that, but you're quite mums are sort of meant to do it all and you know, um I just think that that's quite sad really, that um there's that stigma really that you can't ask for help and you shouldn't, I don't know.”* **Sophie** *: “Yeah and it's always like okay yeah there's still part of you that you don't want to sort of you're like I should be able to do this on my own you know these are my children I should be able to do this but I think we had to quickly come to the realization that it just wasn't going to be that easy.”* **Jennifer** *: “A bit nervy about it at the start because obviously um I I judge a lot of things because obviously my previous experiences…”* **Jennifer** *: “I think it's because there was no Home‐Start there was no real like places to go and get help um there was just no outlet or anything and it's really yeah just really bad.”* **Christina** *: “the lack of support I had from anyone umm my ex, his family that lived down the road. No one, no one helped, didn't know really anyone”* **Jennifer** *: “Um I was I mean my mental health was alright but I would have my down days where I did feel like useless worthless didn't want to do anything really unmotivated and everything but I knew I had to do something to get out from this for my child so yeah.”*
	Taking a leap of faith	**Amanda** *: “After I got into it, I needed to give it a chance and that's the thing you have to give it a chance.”* **Jennifer** *: “Fine yeah um I mean it was at the start it was really daunting I was like oh a new person but then afterwards fine, so yeah.”* **Steven** *: “…how do we know someone's actually going to be helpful coming in here and not an awkward hindrance for us. We weren't sure I think for a little whilst about taking it on…Um until we met [Home‐Start worker] I suppose and began conversations and thought oh she's quite nice and normal.”* **Steven** *: “Yeah and I think I think with Home‐Start as well un until you meet someone from them they're kind of faceless so you just don't know what they're going to be like if you know are they going to send round some strict nanny type figure or whatever you just don't know…until you begin a conversation with them um so at that point of desperation that's when we did ask for them to come round and we were just accepting anything um we really weren't sure but after your first meeting you have quite a great deal of confidence I suppose don't you that it is going to be okay, it will work”*
Having the family in mind	Foundational relationships are built	**Amanda** *: “after about six months it was nanny [volunteer] that's what we called her, on Mother's Day um we've got her like a present and a card to nanny [volunteer]. She was literally like family to us by the end of it and still is now.”* **Christina** *: “…she was amazing um…My, my adopted granny or mum”* **Steven** *: “…she becomes part of the family in some ways doesn't she in terms of it's cuz it's a regular thing consistent and the kids get to know her and stuff”* **Jennifer** *: “She just helped me with obviously my confidence, she helped me with um like putting a routine for [third child] and everything obviously my third child and basically just um and just basically was there to talk to um talk to and everything and it was just really nice just felt like I had a friend.”* **Christina** *: “they're just all little angels…all different but all very good at the same time.”* **Jennifer** *: “Umm it was it was tough it was stressful and yeah when she when she came into the picture it was just so much better and yeah raised my raised my spirits.”* **Amanda** *: “she always bought books…And there was one particular book all the time the Hungry Caterpillar…And she actually go up to [daughter] with a massive note in it before she left as well. Like this is needed someone to look after it…she was was always doing something like or we just talk like and it was always a case of trying to make sure that how're you doing?”* **Sophie** *: “And that's nice it was nice to have that bond where someone you know does does care about if they're developing and yeah and being exciting being excited that they're talking or doing different things um…Cuz yeah she shares in that doesn't she she's been there for the several meetings and”* *Steven: “Yeah absolutely I've not really thought about it but yeah you're right she's quite attached to them herself she's journeyed so much with us”*
	Compassionate understanding	**Amanda** *: “it was like kind of it was like therapy. In a way. I was just having someone there that I didn't want to talk to my mum about. And I didn't want to talk to my partner about…And it was like a safe space with her”* **Christina** *: “…they were just um lovely. All of them. All three of them were amazing and very it was just nice to talk to somebody umm who it was sort of not independent, but, you know, sort of doesn't know your situation doesn't know umm umm doesn't know they just listened, I suppose.”* **Jennifer** *: “Um just basically just the um no no judging and just I could just talk freely and not feel like I was being you know looked down on or anything, it was just so nice and just so she was just so welcoming, yeah.”* **Amanda** *: “it was all on my pace and it can be on your pace. It's it's they're there for you. It's what you're feeling comfortable with. Which is great. Because if it had to be like a certain way, and I don't like that I would have said nope not doing that. But they didn't. I didn't feel anxious with them. And I didn't feel like I had to do something that I didn't want to do”* **Steven** *: “…there's nothing they do to make it awkward or hard”* **Sophie** *: “They just always want to help. There's always opportunities that we're being like [Home‐Start worker] will always say we've got you know we've been approached and asked whether people want to go there was an outdoor er like a forest school kind of thing and they had some free places and so they're always offering passing on offers like that aren't they”* **Sophie** *: “[Home‐Start worker] was really reassuring wasn't she I think it was someone that got it and that we weren't having to‐“* **Steven** *: “She she understood”* **Sophie** *: “battle for getting any help or anything she was yeah just really positive.”* **Steven** *: “And I suppose presenting it as well not in the way of we're trying to impose something on you we're very much a we're here to help, we're flexible, you dictate to us in many ways”* **Steven** *: “I guess it comes at a point where this is exactly what we needed we needed somebody to come who wasn't going to be a pressure on us who was just going to be there to help was happy to do a multitude of things or allow [Mum] to go and do different things if she needed to”*
The butterfly effect of help	The ripple to the children	**Amanda** *: “Because it's just, it's so emotional, I can feel myself getting upset now (tears in eyes). They, they made a massive difference to me. Because of everything that happened. But they made a difference to her. And I don't know what I do if they didn't help with her. Like, the way she is now and that's thanks to them being in my life. And I'll always be grateful for that”* **Sophie** *: “he he's not that comfortable so it's not been that easy having help because actually he wouldn't go to anybody so to have [volunteer] come every week means that he's built up that you know he's comfortable with her and he'll be able to be left with her yeah and she knows what helps and what doesn't so that's way more useful than just somebody that dips in and out doesn't it cuz she actually knows him”* **Jennifer** *: “Lovely. Yeah, yeah. She was really just open arms, cuddles, hugs, yeah just it was just magical yeah. It was lovely so.”*
	The ripple in life and the world	**Amanda** *: “I would love to know what my life would have been like, without Home Start…But I think I'd been poorly. I think I'd still be ill. And I think that my life would be completely different, especially with her. And I don't think she or me would be where I am in this home, owning a car, having a job.”* **Sophie** *: “…like not having [volunteer] and not being able to have that one day a week where we've got the help would just be really hard going wouldn't it I”* **Steven** *: “It would I think probably like it would be a less happy home in many ways as well. You can have fun with the kids with [volunteer] can't you”* ***Sophie**: “Mm”* ***Steven**: “and [volunteer] has fun with the kids as well so it's a positive day whereas perhaps without [volunteer] would have been a grind to get through whatever [indistinguishable word] when she wasn't here”* **Christina** *: “it's a big thing to volunteer for, but for from a mum's perspective it's just it's vital, it's just invaluable to them umm that's why I'd like to do it myself um when they're in school or something”*

### 3.1 Group Experiential Theme 1: Navigating Parenthood

The Group Experiential theme “Navigating Parenthood” captured the complex journey of being a parent, characterized by contrasting emotions and experiences. Parents faced a gap between expectations and reality, leading to significant adjustments. Individual circumstances added unique challenges to the universal aspects of parenting that were challenging enough on their own.

#### 3.1.1 The Contrasting Realities of Parenthood

Parents conveyed the realities of parenthood and its juxtaposition. Their experiences illustrated the coexistence of various emotions such as joy and challenges, struggles and fulfillment. Jennifer found parenthood “challenging but very rewarding at the same time.” For Sophie and Steven, the challenges they faced during parenthood were helped by the sense of joy and appreciation on being parents, with Steven calling it “the greatest priviledge of our life.” This is because the journey to becoming parents was difficult and they faced the possibility of not being able to have children. Sophie conveyed “even the hard times it just makes up for it the fact that we're getting to do this we're getting to be a family.” This highlighted the emotional depth of the journey for the parents as they acknowledged the highs and lows. There was a shared understanding between Sophie and Steven as they validated each other's feelings, showing their emotional connection and the significance of what they had been through.

For Amanda, Sophie and Steven, the expectation of becoming parents differed to the reality. Steven expected an easier adjustment into parenthood where children would “fit into life”. However, this was not the reality. Sophie acknowledged Steven's shock and unpreparedness of the realities of having children. Even with an optimistic view of life, it did not prepare Steven for the challenges that arose and the acknowledgment that the child did not fit seamlessly into their lives as he expected them to. Amanda experienced a contrasting reality between the worry of what she thought would happen when giving birth, compared to the transformative experience that it actually was. She realized the significance of becoming a mother changing her view of life and the child becoming her “absolute world.”

Christina and Jennifer both had a difficult start to becoming parents. Christina's life circumstances contributed to a difficult start into parenthood. She was isolated as she could not drive and struggled to connect to anyone in her local community because there were no other parents with newborn babies. Jennifer also experienced a stressful start to parenthood, calling it “daunting.” She lacked a support network when becoming a mum for the first time meaning she was navigating new parenthood alone. This lack of support intensified the difficulties she was facing and contributed to isolation. She talked about not having the support of family or anyone else at the time, indicating that this would have made a difference to her experiences if she had this support there, also reflective of Christina's experiences of no support.

#### 3.1.2 The Multifaceted Layers of Parenthood

Parenthood was not a singular experience but rather a multi‐layered journey filled with various dimensions, challenges, emotions, and responsibilities. Christina conveyed the additional layers on top of the challenges all parents face in becoming parents, which was reflected in all the other parent's experiences too. All the parents had differing life circumstances occuring on top of the challenges of becoming parents. Amanda experienced the transition to parenthood during COVID‐19. She talked about the significance of becoming a mother for the first time during the pandemic and the reality of this compared to how it could have been had it not been a pandemic. She conveyed feeling “robbed” of her expected experiences and there was a sense of loss for the typical experiences and support she could have had, such as support from her mum.

Parenthood can be challenging enough on its own, yet Christina also struggled with anxiety and depression, which exacerbated the challenges she faced as a new parent. Furthermore, she was in an abusive relationship, exacerbated by COVID‐19 and being stuck in a house with her abuser. This had a profound impact for her, feeling trapped in trauma. The impact of this was still with her. Christina also struggled with the transition of becoming a parent of two and navigating this dynamic, saying that her first daughter's “world was knocked upside down” when her second daughter was born. Christina was trying to help her elder daughter as well as look after a baby as well as navigating the transition not just for herself, but for her elder daughter adjusting to a new family member.

Sophie and Steven experienced the shock of having twins and navigating the unique challenges this brought to parenthood. Although Sophie expressed wanting children, she did not contemplate having twins and what this would mean looking after two babies at the same time, expressing that “it took me a long time to come round to it.” Steven had a differing view where he felt that they could do the same thing they did with their first child, but with two, indicating that the experience would not be too disimilar from what they went through with their first born child. This is interpreted as seeing some humor between Sophie and Steven, perhaps helping them to navigate the realities of twins. Their differing expectations end in the same reality that parenting twins required adjustments beyond duplicating the experiences with one child. Sophie and Steven also navigated the additional layer of a NICU stay for their twins. This could be seen as a tough experience for the parents trying to navigate life and its challenges the best way they could, yet “feeling guilty” if they felt they were not meeting everyone's needs as parents. Sophie and Steven were going through intense challenges and so were not meeting their own needs. Steven says “we were surviving.” Sophie and Steven were giving their all to the children and had no capacity for anything other than caring for them. However, privately, the parents were dealing with immense stress. This shows the hidden difficulties and strains parents can face with their own needs not being met.

Jennifer was navigating an additional layer of parenting a child with autism. She was struggling with understanding what her child wanted because of his lack of communication. Jennifer felt “really anxious” wanting to help her son, yet helpless to know what help her son wanted, leading to more anxiety for Jennifer and the commencement of a vicious circle.

### 3.2 Group Experiential Theme 2: The Process and Significance of Help

This theme reflects the journey parents go through in acknowledging their need for help, reaching out for help, and ultimately embracing it.

#### 3.2.1 The Meaning of Help

Christina, Sophie, Steven and Jennifer conveyed the struggles with acknowledging, asking and accepting help and what this meant to them. Christina reflected on not receiving any support including from family close by, showing the significance and impact of this on her experiences of new parenthood and its navigation with no help: “the lack of support I had from anyone my ex, his family that lived down the road. No one, no one helped, didn't know really anyone.” Christina talked about the difficulties for all mums to reach out for help and, in particular, for new mums if there is no support nearby. She conveyed the stigma associated with asking for help where “mums are sort of meant to do it all and you know, um I just think that that's quite sad really, that um there's that stigma really that you can't ask for help and you shouldn't, I don't know.” There is a societal expectation on mums to carry on without asking for or accepting help. This reflected her own feelings as to why she found it difficult to ask for help.

Finding it difficult to ask for help was also expressed by Sophie, as well as being able to do everything herself. She expressed “I should be able to do this on my own you know these are my children I should be able to do this but I think we had to quickly come to the realization that it just wasn't going to be that easy.” Sophie had an internal struggle between what she felt she “should” be doing and what the reality was. The use of “should” indicated internalized pressures to parent independently. The shift from “I” to “we” suggests a collective realization with Steven about the difficulties of parenthood. The phrase “quickly come to the realization” indicates a sudden confrontation with reality, leading to an acceptance of the complexities of parenting.

Jennifer also struggled with the concept of help, feeling nervous about accessing support. Jennifer had difficult past experiences with support, which had impacted on her judgment when faced with the prospect of accessing help, leading to anxiety of what the help would be like. Futhermore, she had experienced times of no support as a parent referring to there being “no outlet” suggesting no way of releasing stress. She described these times as “just really bad” conveying the profound negative impact of this isolation on her parenting experience. The meaning of help for Jennifer was reflected in her need to make a change for her third born child: “my mental health was alright but I would have my down days where I did feel like useless worthless didn't want to do anything really unmotivated and everything but I knew I had to do something to get out from this for my child.” Although Jennifer was in a bad place mentally, she knew she had to do something to change this. The transformation of parenthood meant recognizing the need to change for her child.

#### 3.2.2 Taking a Leap of Faith

The transition between acknowledging help and then reaching out was expressed by parents. There was a sense of the significance of this step to first accessing support‐ the process of what it meant to access the support. It required a leap of faith, the importance of giving it a go. Amanda expressed the need to give the help (Home‐Start) a chance: “After I got into it, I needed to give it a chance and that's the thing you have to give it a chance.” Once Amanda accessed the help, her feelings towards it changed and she was glad to have given it that chance. She expressed the importance of taking this step.

Jennifer expressed anxiety with accessing the support calling it “really daunting,” particularly surrounding letting a new person (volunteer) into their world. She reflected on the relief that this went well. Steven also expressed the reluctance in accessing help worrying that it could be an “awkward hindrance,” yet the relief in taking this step. There was anxiety around the unknown of what the help from Home‐Start would be like. Sophie and Steven had help offered to them that turned out to be unhelpful and not what they needed. However, on the first meeting with Home‐Start, faith had been restored. This is conveyed further here: “I think with Home‐Start as well un until you meet someone from them they're kind of faceless so you just don't know what they're going to be like…until you begin a conversation with them um so at that point of desperation that's when we did ask for them to come round and we were just accepting anything um we really weren't sure but after your first meeting you have quite a great deal of confidence I suppose don't you that it is going to be okay, it will work.”

### 3.3 Group Experiential Theme 3: Having the Family in Mind

This Group Experiential Theme highlights the pivotal role Home‐Start and the volunteers played in parents' lives, becoming like family members by providing emotional and practical support. These bonds, built on trust and mutual understanding, gave parents a sense of stability during challenging times. Volunteers' compassionate, non‐judgemental approach created a safe space where parents felt understood and empowered, with support tailored to their specific needs, profoundly shaping their journey through the early years of parenthood.

#### 3.3.1 Foundational Relationships Are Built

This subtheme showcases the deeply significant and pivotal role that relationships between the family and volunteer had, with the connection being a foundational pillar in the parents' journey. The volunteers provided emotional and practical support as well as stability during a critical phase of the parent's lives. These relationships served as groundwork upon where trust and understanding were established, creating a solid base that influenced the parents' journey through parenthood.

The impact of the volunteer can be seen when the parents compared the volunteers to members of their family or friends. Amanda called the volunteer “nanny” and described her “like family to us by the end of it.” She said how she got the volunteer a present and card on Mother's Day. This reflected the level of trust and bond that had been formed between the volunteer and the family, where Amanda and her daughter had let the volunteer into their lives. Christina also reflected this notion of the volunteer being like family, calling her “my adopted granny or mum.” This showed how the volunteer held a special place in the parent's life akin to a mother or grandmother figure. The use of “adopted” conveyed a sense of chosen family, implying that this person held immense significance and care in their life. Steven expressed how the volunteer had become a part of the family. The volunteer had become a consistent presence in their lives and was like a member of their family. The children were familiar with her reflecting how comfortable they all felt with her and the bond they all had. Jennifer referred to her volunteer like she “had a friend.” This expressed the depth of the relationship beyond practical assistance. The sense of having someone to talk to, confide in, and share experiences with was immensely meaningful to Jennifer, creating a strong bond.

The volunteers were like bright lights in the parents' lives that provided the right support at the right time, when it was most needed. Christina described the volunteers as “angels.” This was an affectionate statement highlighting how significant the volunteers were, showing they were all different in their approach to help, yet all admirable and helpful evoking a sense of fondness from the parent and how much it made a difference to her life. Jennifer described how her volunteer “raised her spirits.” When the volunteer came into her life, there was a noticeable shift. There was a considerable improvement in her wellbeing and mood, like a weight had been lifted. Amanda conveyed how meaningful the bond with the volunteer was regarding a book the volunteer brought for Amanda's daughter and the significance of this exchange: “she always bought books…And there was one particular book all the time the Hungry Caterpillar…And she actually go up to (daughter) with a massive note in it before she left as well. Like this is needed someone to look after it.” Amanda expressed the thoughtful and caring nature of the volunteer and how she was towards her daughter. The giving of this book held deep significance for Amanda, and the act of leaving a note inside showed the considerate gesture from the volunteer, holding the family in mind and knowing the child's love for this book. It indicated a fondness for the family from the volunteer as well as a fondness from the family for the volunteer, a mutual relationship. Sophie and Steven also expressed the meaningful bond with the volunteer where the volunteer “shares in” the children's development. Sophie and Steven had an appreciation for the care the volunteer had for the family and how the children were doing. They noted that the volunteer had journeyed alongside them, implying a shared emotional connection and involvement in the family's journey and growth.

#### 3.3.2 Compassionate Understanding

Home‐Start and its volunteers provided a safe, supportive, non‐judgemental space. Its foundations were raised on compassion and understanding. Home‐Start always had the family in mind, and this was felt and experienced by the family, instilling hope, faith and the provision of meaningful support. Amanda conveyed the safe supportive space Home‐Start and its volunteer provided, meaning she could openly talk about things that she may not have opened up to family about, describing it like “therapy” and a “safe space.” This was also expressed by Christina who found it helpful to speak to somebody independent of her circumstances who “listened.” This reflected the compassionate understanding of the volunteers felt by the parents, providing that much needed space to talk, without judgment, when the parent most needed it. Jennifer further experienced this non‐judgment from the volunteer and being able to “talk freely” and not “looked down on,” describing the volunteer as “so welcoming.” This suggests the parent felt at ease with the volunteer. There was a sense of acceptance and understanding offered by the volunteer, creating a safe space for open conversation and sharing. It also insinuates that Jennifer has had bad experiences of support in the past where she had felt judged, but that this was different with Home‐Start, providing relief and being able to build a trusting friendship with the volunteer.

The compassionate understanding by Home‐Start and its volunteers was also reflected through their patience and “no pressure” attitude. They tailored the support to what each family needed by listening to the family and understanding their needs in their entirety so that they could provide what could help, without any pressure and led by the family. This is articulated by Amanda when she talked about the volunteer going at her pace, showing a relationship formed on mutual respect: “it was all on my pace and it can be on your pace. It's it's they're there for you. It's what you're feeling comfortable with. Which is great. Because if it had to be like a certain way, and I don't like that I would have said nope not doing that. But they didn't. I didn't feel anxious with them. And I didn't feel like I had to do something that I didn't want to do.” Building trust with the parent helped her to feel comfortable and feel in control of the help received. Sophie and Steven also experienced the no pressure approach of Home‐Start, where Steven says “there's nothing they do to make it awkward or hard” and Sophie saying “they just always want to help.” This reflected Home‐Start always having the family in mind and making the help easier to accept for the family. Keeping the family in mind showed Home‐Start's proactive approach, understanding and dedication to helping families.

Furthermore, the parent's feelings towards Home‐Start convey that Home‐Start understand what it can be like to be a parent and the challenges that they can face and that those challenges can be different depending on each family's circumstances. Sophie and Steven felt that their volunteer also reflected these values that Home‐Start had, where help was provided in a collaborative way where the family felt in control of how they received support. This led Sophie and Steven to feel validated and understood. Steven said: “this is exactly what we needed we needed somebody to come who wasn't going to be a pressure on us who was just going to be there to help was happy to do a multitude of things or allow [mum] to go and do different things if she needed to.”

### 3.4 Group Experiential Theme 4: The Butterfly Effect of Help

The impact of compassion from Home‐Start and a volunteer coming to a family's home to help with what is needed had significant and far‐reaching consequences on the family's life. Through seemingly small ways of helping, it created a chain reaction of positive changes in the family's life trajectory.

#### 3.4.1 The Ripple to the Children

This butterfly or ripple effect was seen by the parents for their children, where they could see the difference the support they were receiving was having for them. Amanda expressed the profound significance of the difference it made for her daughter: “Because it's just, it's so emotional, I can feel myself getting upset now (tears in eyes). They, they made a massive difference to me. Because of everything that happened. But they made a difference to her. And I don't know what I do if they didn't help with her. Like, the way she is now and that's thanks to them being in my life. And I'll always be grateful for that.” The transformation for both mum and daughter was profound and meaningful. The deep sense of gratitude expressed shows the critical role the volunteer had and the bond they formed. Although the support finished, the impact remained with lasting recognition of its positive impact on their lives.

Sophie also expressed the bond formed between the volunteer and their children. One of Sophie and Steven's twins found it difficult to feel comfortable and build trust with others. The mum was grateful for the consistent support from the volunteer and her compassionate understanding and consideration of the child's individual needs. A meaningful bond was formed between the child and the volunteer, providing relief for the mother and indicating the quality of the support provided.

Jennifer described the bond between her son and volunteer as “magical.” She talked about the volunteer in an affectionate way, describing a warm and welcoming experience for herself and her son. The use of the word “magical” reflected the deeply meaningful experience that was had and emotive response to observing the bond between her son and the volunteer and how important and welcoming this must have been for her son.

#### 3.4.2 The Ripple in Life and the World

The wider implications of the help received from Home‐Start and its volunteers was conveyed by the parents. For Amanda, the ripple spread wide: “I would love to know what my life would have been like, without Home Start…But I think I'd been poorly. I think I'd still be ill. And I think that my life would be completely different, especially with her. And I don't think she or me would be where I am in this home, owning a car, having a job.” Amanda reflected on her personal growth and the transformative role Home‐Start had in her life. There was an immense sense of gratitude and personal reflection for the journey she had been on and what it could have been like had she not accessed Home‐Start, showcasing the butterfly effect of the support she received.

Sophie and Steven reflected on the support received and the ripple this had on their lives, with support 1 day a week making a big difference, with Steven saying “it would be a less happy home in many ways as well. You can have fun with the kids with (volunteer) can't you…and (volunteer) has fun with the kids as well so it's a positive day…whereas perhaps without (volunteer) would have been a grind to get through.” Like Amanda, Sophie and Steven reflected on what life would be like had they not had the support from Home‐Start. The volunteer's presence contributed positively to the family's overall wellbeing.

For Christina, the impact of the support received instilled the desire to help others and volunteer herself when it became feasible. She recognized the invaluable role volunteers played in helping families, reflecting her empathy towards other mums. The ripple here was the future, with the lasting impact of the support instilling a drive to help others because of the understanding of the immense value it can have.

## 4 Discussion

The five parents in this study went on a transformative journey from navigating parenthood and its challenges, their feelings towards accessing help and recognizing the need. Accepting this help led to experiencing meaningful compassionate connections with Home‐Start and its volunteers. Foundational pillars were created which catalyzed a butterfly effect across the family and beyond. The results are discussed in relation to previous research, implications for practice and policy and future research.

### 4.1 Understanding the Parent Journey

#### 4.1.1 The Complexity of Early Parenthood

Regarding the first research question in this study on how parents navigate the early years of parenthood, our findings revealed that parents experienced a complex journey characterized by contrasting emotions and expectations (Group Experiential Theme 1: Navigating Parenthood). The juxtaposition of emotions experienced was very apparent, consolidating past findings surrounding the complexity of the transition to parenthood (Cowan and Cowan 2000; Feeney 2001). IPA brought forward the convergences and divergences between the participants. All four cases had differing as well as overlapping circumstances that impacted on their experiences of parenthood, particularly regarding the added layers of parenthood each family was experiencing. Previous research has demonstrated the variety of changes and circumstances that impact parent stress (Cowan and Cowan 2000; Kohn et al. 2012).

This showed what parents' experiences were like of navigating parenthood before accessing Home‐Start Gloucestershire home visiting. In navigating the complexities of early parenthood, our findings resonate with existing literature on Home‐Start impact, which emphasizes the importance of early intervention in reducing parental stress and improving family dynamics (Home‐Start 2014, 2017, 2019, 2021). This connection highlights how support can mitigate the emotional turmoil that many parents face.

#### 4.1.2 Help‐Seeking Process and Barriers

The process and significance of asking for help surfaced across all four cases. The importance of the process and of IPA being a part of exploring this is discussed by Smith et al. (2022). This shows the significance of conducting qualitative research for interventions to understand the process of what parents go through and how best we can support them with accessing support. This feeds into this study's second research question regarding parents' experiences of Home‐Start Gloucestershire home visiting services as well as this study's third research question of what parents' experiences of navigating parenthood were like before accessing Home‐Start Gloucestershire's home visiting service. It was a big step for all parents to take and a very significant one on their journey.

Stigma was a part of this process and has been seen as a barrier to seeking help, yet little is known about the extent to which stigma affects participation in parenting interventions (Alonso‐Marsden et al. 2013; Lanier et al. 2017). Fear of stigma can prevent engagement in parenting interventions. Research has previously found that mothers feared being stigmatized as a “failing” mother and therefore tried to cope alone rather than engage in home visiting (Barnes et al. 2006). The term “home visiting” has been found to put families off due to its perception of being impersonal and judgemental (McInturff et al. 2015). The participants in our study did talk about trying to cope on their own and the stigma on mothers to “do it all,” although there was no mention of a negative perception toward the term “home visiting.”

Conversely, research with fathers has found that they reported low levels of perceived public stigma suggesting that it could be becoming more socially acceptable with changing attitudes towards parent interventions (Lanier et al. 2017). Once the participants had accessed Home‐Start home visiting for the first time, attitudes toward the intervention changed in our study, with parents reflecting on their experiences and the importance of that leap of faith.

Taking the time to facilitate the engagement process and being sensitive to parent's circumstances is important to parents when engaging and starting a parent intervention (Miller and Prinz 2003). Parents should be free to decide whether to participate (Butler et al. 2020). This was reflected in our study with parents appreciating the time and space Home‐Start gave them and allowing the parents to lead. Our analysis sheds light on the stigma that often accompanies the decision to seek help, corroborating findings from the literature that indicate many parents fear being labeled as inadequate (Alonso‐Marsden et al. 2013; Barnes et al. 2006). However, participants in our study reported that their initial reservations dissipated once they experienced the supportive environment fostered by Home‐Start volunteers, aligning with previous findings that highlight the non‐judgmental nature of such programs (McInturff et al. 2015).

### 4.2 The Power of Relationships

#### 4.2.1 Volunteer‐Family Connections

The results highlighted the significance of the relationship between families and volunteers (Group Experiential Theme 3: Having the Family in Mind), contributing to the second research question in this study exploring parents' experiences of Home‐Start's home visiting services. Parents described volunteers as becoming like family, providing both emotional and practical support. The strength of the rapport that parents had with Home‐Start and its volunteers was very prominent in our study. Interactions with more rapport has been shown to be more satisfying to one's needs (Baker et al. 2020).

#### 4.2.2 Non‐Judgemental Support

When comparing the impact of volunteer‐based programs to professional‐based interventions, it is essential to consider the nature of relationships formed during these interactions. Our research aligns with previous studies indicating that volunteer relationships often foster a sense of familial connection (Burn and Almack 2018). Participants expressed that volunteers became like family, providing non‐judgmental support that eased the stigma often associated with seeking help. This emotional rapport is crucial, as previous research suggests that parents may feel intimidated or judged when engaging with professionals, which can hinder their willingness to seek support (Alonso‐Marsden et al. 2013; Lanier et al. 2017). In contrast, professional‐based home visiting programs may often be perceived as more formal and distant, leading to apprehension among parents regarding vulnerability and openness (McInturff et al. 2015). Our findings underscore that the more personal, empathetic interactions characteristic of volunteer‐based programs may enhance parents' engagement and willingness to embrace support, leading to more significant, long‐lasting transformations.

#### 4.2.3 Matching Considerations

However, in our study, we did not ask participants how they felt about having volunteers specifically, and so this is based on our interpretation. Previous research with Home‐Start has shown that parents have found problems with how valuable the support was when there was a mismatch between the volunteer and the parent (MacPherson et al. 2010). None of the parents in our study conveyed a mismatch, although one parent conveyed that all of her volunteers had a different approach, she and all the other parents spoke of the volunteers with a fondness, suggesting that the parents were either matched well with the volunteers or it was not raised as an issue. The staff coordinators of Home‐Start home visiting in another qualitative study reported the need for careful and sensitive matching between the volunteer to the family (Burn and Almack 2018), so perhaps this is what happened with parents and volunteers in our study.

### 4.3 Transformative Impact

#### 4.3.1 Individual and Family Changes

The third research question of our study explored parents' experiences before, during, and after accessing Home‐Start services. Our analysis revealed a transformative journey (Group Experiential Theme 4: The Butterfly Effect of Help), where the impact of Home‐Start's support extended beyond the immediate family context. Parents reported positive changes in their children's and their own wellbeing, showing the far reaching and long lasting effects of the support for them. Our research and the impact of the support received shows the significance of receiving help in the early years and the difference it made to these parents.

#### 4.3.2 Ripple Effects Beyond the Family

The butterfly effect was profound with seemingly small acts of kindness and support having a ripple effect through the family and beyond. With the impact of the polycrisis and the recognition that parenting is too big a task for parents and caregivers to do alone, support is needed to help give children the best possible start in life (UNICEF 2023). This qualitative research has helped to show the meaning and significance of support during the early years identifying the key aspects of change that make interventions meaningful and helpful to families (Furlong and McGilloway 2012; Holtrop et al. 2014; Kane et al. 2007).

#### 4.3.3 Long‐Term Implications

This is a strength of this study, showing the depth of the parents' experiences. The concept of the “butterfly effect” is not only a testament to individual transformation but also aligns with the broader literature on home visiting interventions, which emphasize the far‐reaching benefits of support during critical early years (Holtrop et al. 2014). The supportive relationships built through Home‐Start extend beyond immediate family dynamics, influencing children's wellbeing and fostering resilience that can impact future generations, as supported by existing research (UNICEF 2023).

### 4.4 Limitations

Demographic information was not collected, aside from whether the participants were mothers or fathers. It would be helpful to collect ages of the participants and ethnicity as well as other information to help with understanding the backgrounds of the participants and presentation of the findings. The absence of key demographic information such as educational background, socioeconomic status, employment status, single parent status, and ethnicity limits our ability to understand how these factors may have influenced participants' experiences of parenthood and help‐seeking. For instance, socioeconomic circumstances may affect both the stressors families face and their comfort in accessing support, whilst cultural background could shape attitudes towards help‐seeking and experiences of stigma. Without this demographic context, we cannot determine whether our findings reflect experiences common to particular groups or how demographic factors might influence the transformative journey described. This is particularly relevant given Home‐Start's aim to reach diverse families, and understanding how these influences could inform more targeted service delivery.

The recruitment process was two fold, with Home‐Start Gloucestershire firstly identifying suitable participants. Selection bias could be a factor, relying on staff perceptions. However, the recruitment and sample process chosen helped with building the rapport with the participants and Home‐Start having insight of the parents they support, given the potential vulnerability of parents who have accessed their services. Research was only conducted with participants who live in Gloucestershire, UK. Qualitative research does not seek to be generalizable, so the results cannot be assumed to apply to other parents who have accessed Home‐Start home visiting. However, the in‐depth analysis conducted provided rich data about the parents' experiences (Smith et al. 2022).

The sample size can be considered a limitation of the study. Home‐Start Gloucestershire did identify six families for participation, with four families ultimately participating. Staff reported that it was more difficult than expected to recruit families although the reasons for why this was were not explored. While the idiographic focus of IPA is a strength, offering deep exploration of individual experiences, it may not fully capture the diversity of experiences across broader populations, potentially limiting the generalizability of the findings. However, IPA's primary aim is the detailed examination of personal lived experiences. A key strength of this method lies in uncovering rich, idiographic insights unique to each participant, while also identifying patterns of convergence and divergence across participants' experiences (Smith and Osborn 2008). This reflective and detailed analysis can be time‐consuming, and larger sample sizes can inhibit this approach (Smith et al. 2022). Given the complexity of human phenomena, focusing on a smaller number of participants allows IPA studies to maintain the depth of analysis (Smith et al. 2022). Therefore, the sample size used in this study is appropriate for an IPA approach.

Additionally, this IPA study is part of a broader exploration of Home‐Start services, which utilizes a mixed methods approach incorporating both quantitative and qualitative methodologies. Smith et al. (2022) support the inclusion of IPA within mixed methods research, emphasizing the importance of preserving IPA's core principles in study design. This study fills a gap by providing a detailed account of parents' experiences, capturing the nuances of early parenthood and the meaning of home visiting, including both shared and divergent experiences. By shining a light on this specific area, it may contribute to a broader understanding of the whole. This aligns to Home‐Start's approach where the nature of their support is multi‐faceted and needs based. This can pose a problem for evaluating their programs because different work will be going on with different families. In the United Kingdom, the emphasis is on programs for families with children being evidence‐based, which puts needs‐based programs at a disadvantage (Warner 2018). However, qualitative research helps to bridge this gap alongside quantitative research and specifically, our argument for IPA given its alignment to Home‐Start's family‐centered support that acknowledges the complexities of parenthood including its convergences and divergences.

Two interviews were conducted online (MS Teams) and two were conducted face‐to‐face. In MB's reflective log, they found it easier to conduct face‐to‐face interviews, feeling more atune in the interview space, more able to readily read body language, emotional cues and whether a participant had finished speaking. This aligns to findings regarding the positives of face‐to‐face interviews (Saarijärvi and Bratt 2021). However, travel could be difficult and there are safety risks associated with face‐to‐face interviews. This was mitigated by following risk assessment procedures. During COVID‐19, researchers conducted many interviews online. This technique has become increasingly adopted since the pandemic to help with time constraints and financial burden (Sah et al. 2020). The most important point for our research was to provide participants with the choice of where and when the interview took place, with an insight into what life can be like as a parent and the importance of providing flexibility. Flexibility in qualitative research can improve participant access to research, recruitment, and response rate (Heath et al. 2018).

### 4.5 Implications

This study has implications for practice and policy. In terms of implications for Home‐Start and other national early intervention programs, these findings show the importance of tailoring support to meet the needs of each family, similar to previous findings (Butler et al. 2020). Understanding the process of asking/accepting help can inform Home‐Start's advertisement and engagement with families, as well as informing the referrers of the importance of being sensitive and understanding to family's thoughts and feelings toward accessing support. Furthermore, it highlights the need for all people working with families to understand the complexity of the parent journey and to be mindful of their individual circumstances, as well as being mindful of potential fear of stigma associated with accessing family interventions. Parents are often doing the best they can under extremely difficult circumstances and a non‐judgemental approach from professionals is important (Allen 2011). This is particularly important given that volunteer programs can reach marginalized families who might not engage with formal services, and they provide a model of care rooted in community and empathy (Home‐Start Worldwide 2024).

From a community psychology perspective, this study shows how community‐based interventions can support families who are struggling. The “butterfly effect” identified in this study shows how small acts of community‐based support create far‐reaching positive changes that extend beyond the immediate family. This approach recognizes that many challenges families face stem from broader social and economic factors, including the current polycrisis, and that solutions are most effective when they emerge from and are embedded within the community itself. The volunteer‐based model of Home‐Start home visiting offers an alternative to increasingly strained formal services, creating networks of community support that can reach families who might otherwise remain isolated

This study reinforces the importance of commissioning qualitative research, delving deeper and providing rich data to inform services. This study highlights the importance of the relationship between the volunteer and family. Given the cuts to family services and early help in the United Kingdom (Williams and Franklin 2021), it is critical that services like Home‐Start receive funding to carry on and develop their vital work.

### 4.6 Further Research

This study was based in one county in the United Kingdom. The rich results call for a need for nationwide qualitative research on family early interventions that compliment the more widely used quantitative research, for all Home‐Start services and beyond. Understanding experiences of underrepresented groups is needed, such as interviews with different ethnic communities. In this study, one father was interviewed alongside their partner. Given the lack of the father's voice in research, it is poignant to hear from fathers and understand their experiences (Burgess and Goldman 2022).

Exploring barriers to accessing support is warranted, with an exploration of the process of accessing support and research into stigma in the present day. More research is needed into the impact of the polycrisis for families and how early interventions can be adapted to meet parents' needs. Although research has been conducted regarding the polycrisis itself, there is limited research available on how to navigate it (Rakawski et al. 2025). Recently, there has been some emerging research surrounding the impact of the polycrisis on children and young people (Bessant and Watts 2025; Izydorczyk et al. 2025). However, research around the impact for families remains scarce and there is a need to develop interventions to support families and their wellbeing (Izydorczyk et al. 2025). This includes further research surrounding how home visiting interventions can be adapted given the impact of the polycrisis.

Given the scientific consensus of the importance of the early years of childhood on wellbeing across the life course, longer term impact of early intervention services is needed, including longer term impact of Home‐Start services. Longitudinal studies, including qualitative research, are needed to follow families through their transitions and their children's transitions into adulthood to see what impact Home‐Start home visiting may have had on family wellbeing as time passes.

### 4.7 Conclusion

The early years of parenthood are complex and emotive. It can be difficult yet important for parents to access support during these critical early years. Trusting, non‐judgemental relationships with early intervention services such as Home‐Start can help parents access and benefit from this support. This can lead to a positive impact for parent's wellbeing, children's wellbeing and their lives in general, impacting on the future and across the lifecourse. Qualitative research is critical for understanding the parent journey and helping to adapt early intervention services for the parents' individual needs. It is important to commission qualitative and longitudinal research to inform and provide evidence for funding these vital early intervention services for the future wellbeing of families and our nations.

## Author Contributions

Both authors contributed to the study concept and design. Katerina Kantartzis acquired funding for the study. Material preparation and data collection were conducted by Martha Burlingham. Analysis was conducted by Martha Burlingham and critically discussed, validated and supervised by Katerina Kantartzis. Martha Burlingham drafted the article and Katerina Kantartzis critically reviewed it. Both authors contributed to revisions to the draft before submitting for publication.

## Ethics Statement

This study was approved by the University of Gloucestershire Research Ethics Committee (approval code: REC.23.16.2) on January 13, 2023. All participants provided written informed consent before interviews.

## Conflicts of Interest

The authors declare no conflicts of interest.

## Peer Review

The peer review history for this article is available at https://www.webofscience.com/api/gateway/wos/peer-review/10.1002/jcop.70048.

## Data Availability

The data that support the findings of this study are available on request from the corresponding author. The data are not publicly available due to privacy or ethical restrictions.
